# Immunoturbidimetric determination of serum transferrin on a Kone Progress autoanalyser

**DOI:** 10.1155/S146392468900009X

**Published:** 1989

**Authors:** Luc Cynober, Jacques Le Boucher, Jacqueline Giboudeau

**Affiliations:** Laboratoire de Biochimie A Hôpital Saint Antoine 184, rue du Faubourg Saint Antoine Paris Cedex 12 75571 France

## Abstract

The Kone Progress, a multiparametric discrete analyser, was used to determine serum transferrin with a kit supplied by Kone. Assays recommended by the French Society of Clinical Chemistry were performed in order to assess the suitability of the test. Repeatability was assessed using serum pools with low (L), medium (M) and high (H) concentrations of transferrin. The coeffcients of variation (CV) were 5.4, 3.2 and 2.0% respectively for 30 determinations (within-batch). Reproducibility on 15 consecutive days (between-batch) was also satisfactory (CV for L = 7.3%, M = 6.3% and H = 3.8%). There was no serum-to-serum contamination. Results correlated closely with those obtained using radial immunodiffusion (RID) (r = 0.942) and total iron-binding capacity (r = 0.954)for 90 determinations.

Transferrin measurement by immunoturbidimetry on the Kone Progress emerges as a well-suited, rapid and inexpensive alternative to other time-consuming (RID) and sophisticated (laser immunonephelemeter) techniques.


Journal of Automatic Chemistry Vol. 11, No. 1 (January–February 1989), pp. 40–41

### Immunoturbidimetric determination of serum transferrin on a Kone Progress autoanalyser†

**Luc Cynober, Jacques Le Boucher and Jacqueline Giboudeau**

*Laboratoire de Biochimie A, Hôpital Saint Antoine, 184, rue du Faubourg Saint Antoine, 75571 Paris Cedex 12, France*

*The Kone Progress, a multiparametric discrete analyser, was used to determine serum transferrin with a kit supplied by Kone. Assays recommended by the French Society of Clinical Chemistry were performed in order to assess the suitability of the test. Repeatability was assessed using serum pools with low (L), medium (M) and high (H) concentrations of transferrin. The coefficients of variation (CV) were 5·4, 3·2 and 2·0% respectively for 30 determinations (within-batch). Reproducibility on 15 consecutive days (between-batch) was also satisfactory (CV for L = 7·3%, M = 6·3% and H = 3·8%). There was no serum-to-serum contamination. Results correlated closely with those obtained using radial immunodiffusion (RID) (r = 0·942) and total iron-binding capacity (r = 0·954) for 90 determinations.*

*Transferrin measurement by immunoturbidimetry on the Kone Progress emerges as a well-suited, rapid and inexpensive alternative to other time-consuming (RID) and sophisticated (laser immunonephelemeter) techniques.*

### Introduction

The measurement of serum transferrin is recognized as an indispensable parameter in the exploration of iron metabolism and its measurement is recommended to replace total iron binding capacity (TIBC), the old indirect method, for the evaluation of transferrin in serum [1]. Furthermore, serum transferrin is considered to be a good marker of chronic and semi-acute malnutrition [2] and is a useful parameter in following the nutritional status of patients receiving nutritional support [3].

Transferrin can be assessed by numerous methods, including radial immunodiffusion, laser nephelemetry and immunoenzyme assay [1, 4 and 5]. All are expensive and/or time-consuming. Recently, an immunoturbidimetric assay technique has been described for transferrin [6 and 7] as for other specific proteins such as haptoglobin [7 and 8]. This method is rapid and economic.

In this work the efficiency of the measurement of serum transferrin has been evaluated by immunoturbidimetry using a discrete analyser, the Kone Progress, routinely used for biochemical assays in the Laboratoire de Biochimie A, at the Hôpital Saint Antoine.

† *This work is dedicated to Marie-Thé Gaubert (1962–1988).*

### Materials and methods

*Immunoturbidimetry* was performed using the transferrin test kit (Kone, Finland) containing the anti-transferrin anti-serum and polyethylene glycol to enhance the immunoprecipitation. The apparatus (Progress, Kone, Finland) [9] was used under the conditions described in table 1.

*Table 1. Immunoturbidimetric determination.*

| | | |
|---|---|---|
| Sample | = | 5 µl (1/11 diluted) |
| Buffer | = | 100 µl |
| Incubation | = | 150 s |
| Antibody | = | 30 µl |
| Incubation | = | 120 s |
| Measurement | = | end point |
| λ | = | 340 nm |
| T° | = | +37°C |

The non-linear calibration curve was obtained using the protein calibration set (supplied by Kone) containing transferrin at five known concentrations (from 1 to 8 g/l). Samples of serum and calibrators were diluted at 1/11 before use.

*Radial immunodiffusion:* nor-partigen plates were used (Behring, Marburg, FR Germany) with the appropriate serum control (serum RDT, Behring).

*Total iron-binding capacity* was measured by the classic addition of $FeCl_3$ to serum, elimination of excess iron by $MgCO_3$ and measurement of total bound iron by the bathophenanthroline reaction. Results are expressed in µmol iron/l.

### Results

Within-run and between-run precision was tested with pools of serum from patients at the hospital: low (L), medium (M) and high (H) transferrin concentrations (different pools were used for within-run and between-run assays). The within-run assay consisted of 30 consecutive measurements for each pool; and the between-run of measurements on 15 consecutive days. Results are given in table 2.

Sample dispenser carry-over assessment was performed measuring (10 times) a sample with low transferrin ($L_1$), and (10 times) a sample with high transferrin ($H_1$) and then performing the sequence $H_2H_3L_2L_3$ six times where $H_2$ is the sample $H_1$ measured in first position, $H_3$ is the

0142–0453/89 $3.00 © 1989 Taylor & Francis Ltd.

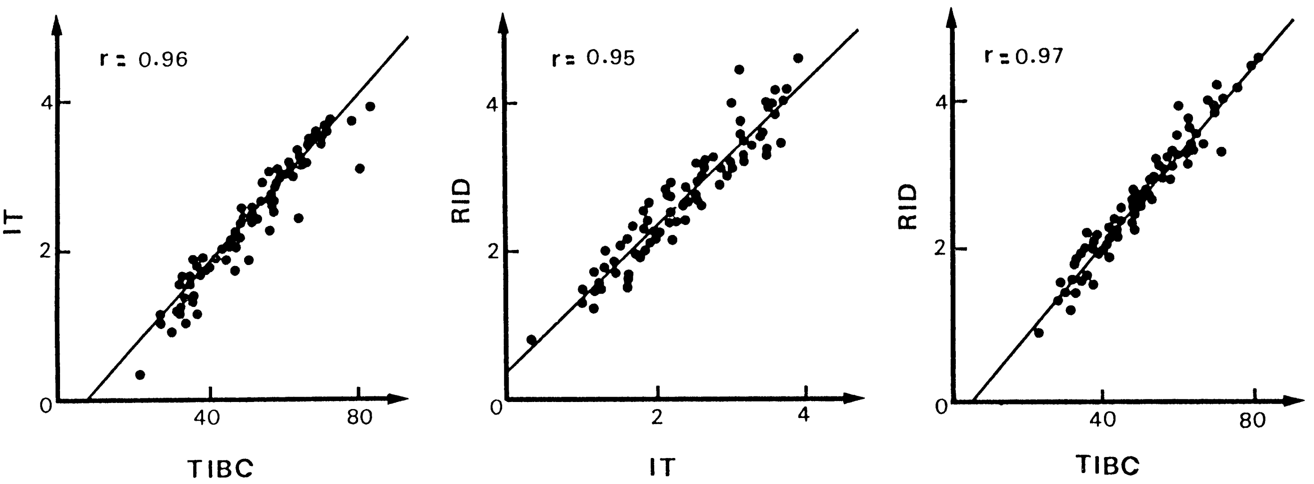

*Figure 1. Correlations between immunoturbidimetry (IT), radial immunodiffusion (RID) and total iron binding capacity (TIBC), on 90 samples from hospital patients.*

*Table 2. Precision assay.*

| | Mean | SD | CV % |
|---|---|---|---|
| *Within-run* | | | |
| L | 0·32 | 0·04 | 5·4 |
| M | 2·33 | 0·07 | 3·2 |
| H | 3·60 | 0·07 | 2·0 |
| *Between-run* | | | |
| L | 1·31 | 0·10 | 7·3 |
| M | 2·47 | 0·16 | 6·3 |
| H | 3·19 | 0·12 | 3·8 |

sample $H_1$ measured after $H_2$, $L_2$ is the sample $L_1$ measured after $H_3$ and $L_3$ is the sample $L_1$ measured after $L_2$. Mean ± SD are then calculated for $L_1$, $L_2$, $L_3$, $H_1$, $H_2$ and $H_3$. There was no significant difference between $H_1$ and $H_2$, $H_2$ and $H_3$, $L_1$ and $L_3$ (data not shown).

The method comparison was performed using 90 patient sera, which were analysed by TIBC determination, RID and immunoturbidimetry (IT) (results expressed in g/l). Results are presented in figure 1 and in table 3.

### Discussion

Transferrin has been assessed indirectly by the measurement of TIBC for some years. However, there is now a general consensus [1] that advances in clinical biochemistry make the direct determination of transferrin preferable to TIBC. The most common methods are IDR and laser nephelometry; but the former takes 48 hours and the latter requires expensive, dedicated apparatus. Immunoturbidimetry thus appears as an attractive alternative, especially when automatized on a routine analyser. Immunoturbidimetric determination of transferrin on the Kone Progress proved to be accurate, reliable and inexpensive. It is interesting to note that immunoturbidimetry gave significantly lower values than RID ($T = 27{\cdot}4$; $p < 0{\cdot}001$). There is no clear explanation for this difference but this has previously been reported

*Table 3. Comparison of serum transferrin measured by immunoturbidimetry (IT) and radial immunodiffusion (RID) or assessed by total iron binding capacity (TIBC).*

| Comparison | *N* | Slope and intercept ($y = ax + b$) *a* | *b* | *r* |
|---|---|---|---|---|
| IT/TIBC | 90 | 0·057 | −0·46 | 0·96 |
| RID/IT | 90 | 0·977 | 0·36 | 0·95 |
| RID/TIBC | 90 | 0·059 | −0·27 | 0·97 |

[6]: in this work, as in our own, there is a systematic and constant difference in values (intercept different from 0, slope near 1). In the same way, immunoturbidimetry gives lower values than laser nephelometry [4]. These differences requires that the values obtained be compared to normal values for a given technique: normal values of serum transferrin range from 2·50 to 3·50 g/l using immunoturbidimetry [4 and 7].

### References

1. Dezier, J. F., *Annales de Biologie Clinique*, **44** (1986), 583.
2. Carpentier, Y. A., Barthel, J. and Bruyns, J., *Proceedings of the Nutrition Society*, **41** (1982), 405.
3. Fletcher J. P., Little, J. M. and Guest, P. K., *Journal of Parenteral and Enteral Nutrition*, **11** (1987), 144.
4. Plomteux, G., Charlier, C., Albert, A., Farnier, M., Pressac, M., Vernet, M., Paris, M., Dellamonica, C. and Dezier, J. F., *Annales de Biologie Clinique*, **45** (1987), 622.
5. Guindi, M. E., Skikne, B. S., Covell, A. M. and Cook, J. D., *American Journal of Clinical Nutrition*, **47** (1988), 37.
6. Leonberg, B. L., Crosby, L. O. and Buzby, G. P., *Journal of Parenteral and Enteral Nutrition*, **11** (1987), 74.
7. Viedma, J. A., Iglesia, A. and Navarro, A., *Clinical Chemistry*, **33** (1987), 1257.
8. Lee, C. and Boss, M., *Clinical Chemistry*, **33** (1987), 1466.
9. Cynober, L., Morgant, G., Morelet, L. and Giboudeau, J., *Clinical Chemistry*, **33** (1987), 123.

